# Real-World Outcomes of Hodgkin Lymphoma: A Multi-Centric Registry From India

**DOI:** 10.3389/fonc.2021.799948

**Published:** 2022-02-11

**Authors:** Dinesh Bhurani, Reena Nair, Senthil Rajappa, Suparna Ajit Rao, Nithya Sridharan, Rakesh Reddy Boya, Ganapathi S. Raman, Hari Menon, Arun Seshachalam, Ramesh Nimmagadda

**Affiliations:** ^1^ Department of Haematology, Rajiv Gandhi Cancer Institute and Research Centre, New Delhi, India; ^2^ Department of Haematology, Tata Medical Centre, Kolkata, India; ^3^ Department of Medical Oncology, Basavatarakam Indo American Cancer Hospital and Research Institute, Hyderabad, India; ^4^ Department of Medical Oncology, P. D. Hinduja Hospital and Medical Research Centre, Mumbai, India; ^5^ Department of Medical Oncology, VS Hospitals, Chennai, India; ^6^ Department of Medical Oncology, Mahatama Gandhi Cancer Hospital and Research Center, Visakhapatnam, India; ^7^ Department of Medical Oncology, Kumaran Hospital Private Ltd., Chennai, India; ^8^ Department of Medical Oncology, CyteCare Cancer Hospitals, Bengaluru, India; ^9^ Department of Medical Oncology, GVN Hospital, Trichy, India; ^10^ Department of Medical Oncology, Apollo Cancer Institute, Chennai, India

**Keywords:** Hodgkins, prognosis, outcomes, real-world evidence (RWE), Middle Income Countries (MIC), lymphoma-diagnosis

## Abstract

**Background:**

Hodgkin’s lymphoma (HL) is one of the most curable malignancies with a 5-year survival of over 80%. Most published literature from low-middle income countries comes from single institute experience.

**Methodology:**

The OncoCollect Lymphoma group registry was set up in 2017 and has 9 major participating sites across India. Data of newly diagnosed classical HL (CHL) patients, treated between 2011 and 2017, were collected using OncoCollect software. The clinical features, subtypes, prognostic stratification, treatment patterns, response to first-line treatment, and 5-year outcomes were analyzed. All statistical analysis was done using Microsoft R Open statistical software linked to OncoCollect software.

**Results:**

There were 939 newly diagnosed CHL patients with a median age of 38 (range, 18–99) years at presentation. The male-to-female ratio was 2.07:1. Histological subtypes included mixed cellularity, CHL (MC, CHL), nodular sclerosis, CHL (NS, CHL), lymphocyte-rich, CHL (LR, CHL), and lymphocyte-depleted, CHL (LD, CHL), in 60.60%, 26.94%, 9.80%, and 2.66%, respectively. At presentation, 50.43% had B symptoms and 53.35% had advanced disease. 29.71% of advanced-stage patients had high Hodgkin IPI score. 79% and 21% of patients received 1st-line treatment with chemotherapy alone or combined modality treatment with chemotherapy and radiotherapy. The most common first-line chemotherapy was ABVD-based regimen (94.68%). The overall response rate was 93.48%. Complete response rates among early-stage favorable and unfavorable risk groups were 92.73% and 86.79%, and those among advanced-stage low- and high-risk groups were 76.64% and 69.78%, respectively. The median relapse-free follow-up duration was 51 months (IQR 22–69). A significant difference was found in 5-year EFS between the early- and advanced-stage disease 83.53% and 73.55% (p = 0.00087), respectively. Similarly, significant difference was found in EFS among early-stage patients treated with a combination of 4-cycle chemotherapy and radiotherapy vs. chemotherapy alone 88.57% and 66.33% (p = 0.0042), respectively.

**Conclusions:**

In this large cohort from India, survival of patients with HL was comparable to the developed world. With a median follow-up of 51 months, the 5-year EFS and OS of all patients were 78.24% and 83.63%, respectively.

## Introduction

Long-term outcomes of Hodgkin lymphoma (HL) have been reported from single-center retrospective studies from India (1–4). The initial progress in the outcomes of HL can be attributed to better staging facilities, more consistent implementation of standard combined modality treatment, and the wider use of high-dose chemotherapy with autologous stem cell rescue in relapsed disease (5, 6). As long-term outcomes improved and prognostic factors were identified, the emphasis shifted to de-escalating treatment protocols in order to minimize treatment-related morbidity, especially long-term toxicity (7). There is only limited information available regarding these aspects of treatment from resource-limited countries with India contributing to these data in a modest way (5, 8, 9). The ethnic diversity, cultural spectrum differential access to healthcare, and paucity of awareness for cancer and lymphoma in particular make it important to have uniform data collated and analyzed systematically. Given that lymphoma patients are mostly treated in tertiary cancer centers, specialty hospitals, and academia with uniform protocols, data from these centers were captured. The OncoCollect Lymphoma Registry was set up in 2017 to address the previous challenges in collecting retrospective data largely at a time when chart reviews were the main data source. Enrollment of member institutes into the registry is by invitation. OncoCollect is installed in individual institutions. Participating institutions are given the rights to export data, and the OncoCollect exports anonymized data to the Registry. Both academic and community practices are a part of the registry. Every effort is made to ensure that the data are representative in terms of geography, socioeconomic status of patients, and clinical expertise of the institutes. Each institute is responsible for entering data onto the OncoCollect software developed by the Ramesh Nimmagadda Cancer Foundation (RNCF). RNCF is a charitable trust that facilitates collaborative data collection across various cancer subtypes from institutes in India. The software has the ability to import data from the hospitals electronic medical records (EMR) or Excel sheets, apart from the manual entry. The statistical analysis software is linked to the OncoCollect software.

This study is a collaborative effort to evaluate current practices in the management of HL in a middle-income country, identify the challenges in data collection, and look at potential solutions for future patient management.

## Objective

The primary objective of this study was to analyze the clinical presentation and outcomes for HL in real-world collaboration.

The secondary objective were to study the treatment patterns based on prognostic risk stratification.

## Methodology

This was designed as a retrospective, multi-institutional, observational study to report clinical features at presentation and analyze outcomes of patients diagnosed between January 2011 and December 2017. A total of 1,285 patients (≥18 years) with WHO classification of HL were registered in the database. 939 treatment-naive classical HL (CHL) patients were considered evaluable for first-line treatment response and outcome. 51 patients with nodular lymphocytic predominant HL and 295 patients who presented post relapse or had less than 4 visits in the outpatient clinic with no definite treatment prescribed at the participating centers were excluded from this audit. The electronic medical records (EMR) capturing data of patient characteristics, diagnosis, admission for toxicity, medications, imaging, and laboratory were utilized for this study. This study was approved by the Hospital Ethics Committee (HEC) of all participating institutes. A consent waiver was granted by the HEC.

OncoCollect software developed by the Ramesh Nimmagadda Cancer Foundation (RNCF) was used to collate data.

### Disease Assessment

The histopathological diagnosis was reviewed at the participating center for most patients [subject to slide and block availability] prior to the start of therapy. CHL subtyping was done according to the WHO 2008 (10) and staging and prognostication using the EORTC (11) and NCCN guidelines (12).

Clinical variables recorded from the EMR included age, gender, Eastern Cooperative Oncology Group [ECOG] performance status [PS], fever [>38.6°C], weight loss [>10% of body weight in 6 months], Ann Arbor stage, and preexisting comorbidities. As part of the staging evaluation, whole-body FDG PET-CT or plain CT imaging of the thorax and abdomen along with bone marrow aspiration and biopsy was done. Cerebrospinal fluid (CSF) cytology at diagnosis was done for patients with symptoms or signs of central nervous system (CNS) involvement. Laboratory test results included absolute blood counts, creatinine, albumin, and LDH. Early-stage patients were classified as favorable risk and unfavorable risk (13). The Hodgkin International Prognostic Index [IPI] on the basis of the following criteria (i.e., Hb, age, sex, stage, white cell count, lymphocyte count, and serum albumin) was calculated for patients with advanced-stage disease (14).

### Treatment and Toxicity

The choice of therapy was dependent on the patient’s general condition, comorbidities, available financial, and social support. ABVD (Adriamycin, bleomycin, vinblastine, and DTIC) was the standard regimen used. Elderly patients and those with cardiac or pulmonary comorbidities were given other regimens: COPP (cyclophosphamide, vincristine, procarbazine, and prednisolone) and modifications of ABVD regimens with replacement of Adriamycin or bleomycin by etoposide. Chemotherapy cycles were repeated every 28 days for 4 to 6 cycles, depending on stage and physician’s choice.

Early-stage or limited-stage (stage I and II) patients received 4 to 6 cycles of chemotherapy. Radiotherapy was given to patients receiving less than 6 cycles at the discretion of the treating physician. All advanced-stage (stage III and IV) patients were planned for 6 cycles of therapy followed by radiotherapy to the site of initial bulky tumor [a single nodal mass of 10 cm or more or mediastinal bulk more than one-third of the transthoracic diameter] or for partial response at the discretion of the treating team.

Treatment-related toxic effects reported in EMR leading to hospitalization were analyzed.

### Response and Follow-Up

The efficacy of treatment was assessed according to the revised response criteria of the International Harmonization Project on lymphomas (15). Patients who died on treatment, stopped treatment due to Grade 4 morbidity, or were lost to follow-up prior to mid-cycle evaluation were considered non-evaluable for response assessment. For patients who progressed on treatment or stopped follow-up for any reason post-mid-cycle assessment, the mid-treatment response is reported. The end of treatment response is reported for all patients who completed treatment.

The follow-up of each patient was obtained from the EMR records, or by keeping close contact with the patient/family on phone. Detailed physical examination, blood counts, and ESR were repeated on follow-up visits. Imaging studies for surveillance were as per the institute policy. Patients with residual disease and following relapse after first-line therapy were offered salvage therapy followed by high-dose chemotherapy as consolidation when financially feasible.

Reasons for death have been classified in three groups: progressive disease, treatment toxicity, and other causes. Patients in remission were censored at the last follow-up. Patients with progression and no follow-up information (physical/telephonic) were considered deceased.

### Statistical Analysis

Descriptive analysis was undertaken. Continuous variables are summarized as median and interquartile distance. Categorical variables are expressed as absolute and percentage frequencies. Categorical covariates are compared using the Chi-Square test. The prognostic effect of covariates has been estimated using the Cox proportional hazard regression model and reported as hazard ratio (HR) with 95% confidence interval (CI). The survival functions have been calculated and plotted using the Kaplan–Meier method, and the survival rate at 5 years of follow-up was reported with the estimated 95% confidence interval (95% CI). Overall survival (OS) was defined as the time from diagnosis until death or the end of follow-up. Event-free survival (EFS) was defined as time from diagnosis until progression or relapse, death from any cause, or end of follow-up (August 2021; censoring).

## Results

**Figure 1:**
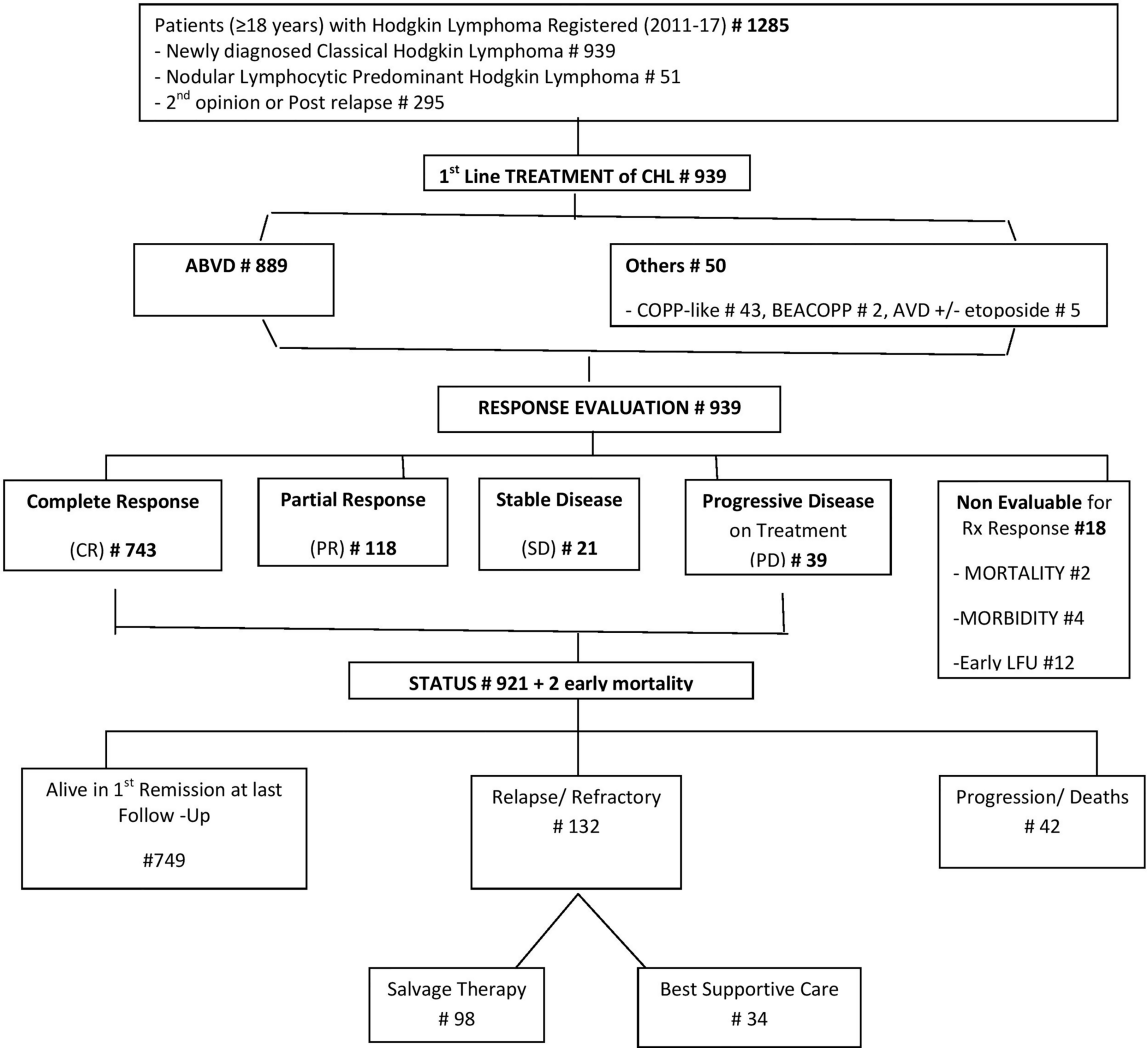
Consort diagram.

HL patients registered at the 9 participating centers are depicted in **Figure 1**. The presenting clinical characteristics of 939 CHL patients are summarized in **Table 1**. The median age was 38 years (18–99). 318 (33.86%) patients were in the second and third decades of their life. The gender ratio was 2.07:1. B symptoms were present in 473 (50.43%) patients. The commonest WHO subtype was mixed cellularity in 569 (60.60%) followed by nodular sclerosis in 253 (26.94%), lymphocyte rich in 92 (9.80%), and lymphocyte depleted in 25 (2.66%).

**Table 1:** Clinical characteristics at presentation, treatment, and response of 939 patients with classical Hodgkin lymphoma.

	Number [N = 939]	Percent [%]
**Age** Median (range)	38 years (18–99)	
<50	664	70.71%
≥50	275	29.29%
**Gender**: ratio	2.07:1	
Male	634	67.52%
Female	305	32.48%
**Stage**		
1	107	11.40%
2	331	35.25%
3	274	29.18%
4	227	24.17%
**B symptoms**		
Absent	465	49.57%
present	473	50.43%
Missing	1	
**Early-stage groups**		
Favorable	57	13.04%
Unfavorable	380	86.96%
Missing	1	
**Late-stage IPI score**		
Low 0–3	336	70.29%
High 4–7	142	29.71%
Missing	23	
**Comorbidities**		
None	539	81.30%
1 or more	124	18.70%
Missing	276	
**HL subtype**		
Mixed cellularity	569	60.60%
Nodular sclerosis	253	26.94%
Lymphocytic rich classical HL	92	9.80%
Lymphocytic depleted classical HL	25	2.66%
**Treatment**		
ABVD	889	94.68%
Others [COPP, AVD+/- etoposide]	50	5.32%
**Chemotherapy-RT**		
Chemo + RT	197	21.00%
Chemo only	741	79.00%
RT only	1	
**Chemo cycles + RT**		
4 cycles	143	15.98%
4 cycles + RT	68	7.60%
6 cycles	557	62.23%
6 cycles + RT	127	14.19%
**Chemo cycles in the early stage**		
4 cycles	149	35.14%
6 cycles	275	64.86%
Missing	14	
**RT in the early stage**		
Yes	130	29.68%
No	308	70.32%
**Response**		
Complete response	743	80.67%
Partial response	118	12.81%
Stable disease	21	2.28%
Progression on treatment	39	4.23%
Non-evaluable	18	
**First relapse/progression**		
Early stage	46	34.85%
Advanced stage	86	65.15%

RT, radiotherapy.

Staging evaluation was done with whole-body FDG PET-CT or plain CT of the thorax and abdomen. Bone marrow biopsy was done in 692 (74.25%). At presentation, 438 (46.65%) had early-stage (1 and 2) and 501 (53.35%) late-stage (3 and 4) disease. In the early-stage patients, 57 (13.04%) were in the favorable group and 380/437 (86.96%) belonged to the unfavorable group. In patients with advanced-stage disease, 336/478 (70.29%) had a low-risk IPI score of 0–3 and 142 (29.71%) had a high-risk IPI score of 4–7.

Documentation of comorbidities was available in 663 EM records. One or more comorbidities were present in 124/663 (18.70%). Diabetes was the most common comorbidity in 69 (10.41%), followed by hypertension in 36 (5.43%), hypothyroidism in 11 (1.66%), chronic obstructive lung disease in 2 (0.3%), and ischemic heart disease in 9 (1.36%). Past history or coexistent tuberculosis was reported in 24 (3.62%). HBsAg, HCV, and confirmed HIV were reported in 6 (0.90%), 3 (0.45%), and 5 (0.75%), respectively.

In univariate analysis, age, stage, B symptoms, and IPI for advanced stage had an impact on EFS but gender, comorbidities at presentation, and early-stage favorable versus unfavorable risk group did not. Inferior EFS was seen in patients ≥50 years (p = 0.0093). However, age was not a significant predictor for EFS in patients treated with ABVD **(** **Table 2** **)**.

**Table 2:** 5-year outcomes of classical Hodgkin lymphoma.

	5-year EFS N = 939	95% confidence interval	p value
**Age group**			
<50 years	80.21%	76.91%–83.65%	0.0093
≥50 years	73.26%	67.61%–79.38%	
**Gender**			
Male	77.04%	73.42%–80.84%	0.64
Female	80.44%	75.79%–85.38%	
**Stage**			
I	82.13%	74.41%–90.65%	0.<00019
II	83.96%	79.75%–88.39%	
III	78.94%	73.61%–84.65%	
IV	67.56%	61.21%–74.58%	
**Early stage**			
Favorable	84.45%	75.05%–95.03%	0.94
Unfavorable	83.60%	79.58%–87.62%	
**Advanced-stage Hodgkin’s IPI**			
Score 0–3	76.56%	71.63%–81.84%	0.013
Score 4–7	65.48%	57.47%–74.61%	
**Comorbidities**			
No	78.27%	74.48%–82.24%	0.068
Yes	73.71%	65.80%–82.57%	
**B symptoms**			
No	83.16%	79.39%–87.10%	0.00032
Yes	73.53%	69.30%–78.03%	
**Stage groups**			
Early	83.53%	79.80%–87.44%	0.00087
Advanced	73.55%	69.33%–78.02%	
**Treatment groups**			
ABVD Others [COPP, AVD+/- etoposide]	79.97%	77.08%–82.97%	<0.0001
	47.94%	35.24%–65.22%	
**ABVD in age group**			
<50	80.86%	77.57%–84.30%	0.16
>50	77.33%	71.49%–83.64%	
**Chemotherapy - RT**			
Chemotherapy + RT	75.85%	69.83%–82.39%	0.67
Chemotherapy only	79.00%	75.75%–82.39%	
**Chemotherapy - RT in the early stage**			
4 cycles	66.33%	55.67%–79.03%	<0.0001
4 cycles + RT	88.57%	80.92%–96.93%	
6 cycles	89.67%	85.26%–94.31%	
6 cycles + RT	77.58%	67.36%–89.35%	
**Interim PET groups**			
CR	90.72%	86.69%–94.95%	<0.0001
No CR	69.30%	60.97%–78.76%	
**End of 1^st^ line**			
CR	88.85%	86.34%–91.44%	<0.0001
No CR	35.38%	27.38%–45.71%	
**5-year EFS**	78.24%	75.36%–81.24%	
**5-year OS**	83.63%	80.86%–86.49%	
**Second line-EFS**	60.66%	49.62%–74.15%	

RT, radiotherapy; CR, complete response; EFS, event-free survival; OS, overall survival.

On multivariate analysis for EFS, stage, B symptoms, and number of chemotherapy cycles were significant. In early disease B symptoms and number of chemotherapy cycles and in advanced disease number of chemotherapy cycles and radiotherapy were significant factors **(** **Table 3** **).**

**Table 3:** Multivariate analysis of all the patients.

Parameter	Coef	HR	Lower CI	Upper CI	p value
**Age**	0.008	1.008	0.998	1.018	0.133
**Ann Arbor stage**	0.338	1.403	1.165	1.688	<0.001
**B symptoms**	0.446	1.561	1.085	2.247	0.016
**Chemotherapy cycles**	-1.119	0.327	0.229	0.465	<0.001
**Radiotherapy**	-0.003	0.997	0.688	1.445	0.988
**Histology subgroups**	-0.048	0.953	0.797	1.139	0.596

### Treatment Efficacy

A total of 889 (94.68%) received ABVD chemotherapy, and 50 (5.32%) received other combinations. The median number of chemotherapy cycles received was 6 (range 1–6). Consolidation radiotherapy was given to 130/438 early-stage (29.68%) and 67/501 advanced-stage (13.37%) patients. CR was achieved in 743 patients (80.67%), PR in 118 (12.81%), and stable disease in 21 (2.28%). 39 (4.23%) had PD on first-line treatment. 18 patients could not be evaluated for response due to early mortality (n = 2), severe morbidity resulting in treatment dropouts (n = 4), or failure to take treatment for financial and other social reasons (n = 12).

Early-stage patients received 4 cycles of ABVD with or without radiotherapy in 64 (15.0%) and 85 (20.0%), respectively, or 6 cycles chemotherapy in 210 (49.5%) and 6 cycles followed by consolidation radiotherapy in 65 (15.0%). The 5-year EFS for favorable versus unfavorable early-stage CHL was 84.45% versus 83.60% (p = 0.94), respectively. Treatment with chemotherapy combinations less intensive than ABVD (i.e., COPP, AVD+/- etoposide) was associated with inferior survival. The 5-year EFS was 79.97% for ABVD versus 47.94% for other combination therapy (p < 0.0001). The 5-year EFS was significantly inferior in advanced- (73.55%) compared with early-stage (83.53%) disease (p = 0.00087).

**Figure 2:**
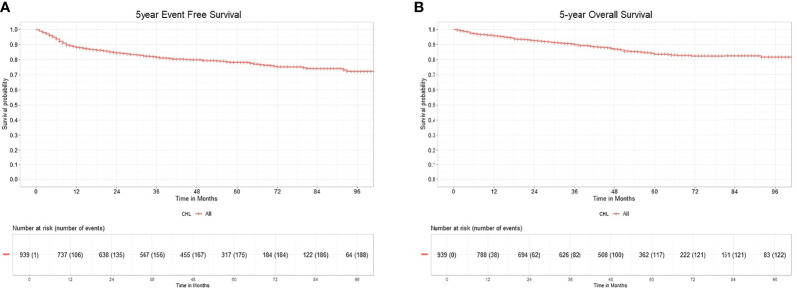
**(A)** 5-year event free survival of classical Hodgkin lymphoma. **(B)** 5-year overall survival of classical Hodgkin lymphoma.

A PET-CT scan for mid-treatment response evaluation was done in 346 patients (36.85%), and an end-of-treatment PET-CT was done in 427 (45.47%). The remaining patients had an evaluation done with CT scans. Inability to achieve a CR on mid-cycle PET-CT assessment showed inferior 5-year EFS, 69.3% versus 90.2%. Patients who achieved a CR at the end of treatment had 5-year EFS of 88.85% as compared to patients who never achieved a CR (5-year EFS 35.38%), as shown in **Table 2**. The EFS for the entire cohort at 5 years is 78.24% (CI 75.36%–81.24%), as shown in **Figure 2A**. The median FU was 51 months (IQR 22–69).

12 patients (1.2%) developed febrile neutropenia and required hospital admission for the same.

Out of 921 patients who completed therapy, 132 patients relapsed or did not respond to first-line therapy. Among relapsed or refractory patients, 98 (74%) patients underwent second-line therapy. Treatment was escalated to BEACOPP or ABVD in 9 patients. Platinum-based chemotherapy was the commonest in 58 (60%), followed by gemcitabine at 26 (27%) as salvage regimen. Only 8 (8%) patients received novel immunotherapies, brentuximab (n = 3), nivolumab (n = 1), and rituximab (n = 4). 15 (15%) patients received metronomic therapy with a combination of cyclophosphamide, etoposide prednisolone, and procarbazine along with best supportive chemotherapy because of advanced age, associated comorbidities, or socioeconomic reasons. 22 (23%) patients underwent autologous transplantation after achieving adequate response. The 5-year EFS for 2^nd^-line treatment in 98 patients were 60.66% (CI 49.62%–74.15%). The median relapse-free survival for the patients with salvage therapy is 28 months (IQR 9.5–51). The OS for the entire cohort at 5 years was 83.63% (CI 80.86%–86.49%), as shown in **Figure 2B**.

EFS events in the first year were very high at 11.5% compared to next 4 years at 3.09%, 2.24%, 1.17%, and 0.85%. This was partly due to patients who discontinued treatment during the first year. Loss of follow-up was 10.54% and 5.75% in the first and second years post chemotherapy respectively with lower numbers in years 3–5 at 3.94%, 3.30%, and 3.73%.

## Discussion

The OncoCollect data represent the largest retrospective series (**Table 4**) of CHL patients treated in India. The important observations from previous studies in India included young age at diagnosis, higher male-to-female ratio, higher frequency of B symptoms, larger proportion of patients with advanced stage, and mixed cellularity being the predominant histological subtype. While the median age of presentation from India has always been lower in earlier series, this study showed median age of 38 years, very similar to that reported in the SEER data (18). Most patients were in their second and third decades of life with no second peak identified after 50 years, a departure from the trend noted from Western countries (14, 19). The absence of a second peak in our study may be explained by the overall younger population of India and reluctance of older patients to go through chemotherapy. This might improve in coming years due to improving financial conditions, social attitude toward chemotherapy, and aging population.

**Table 4:** Comparison of Hodgkin lymphoma outcomes from Indian studies with SEER data.

Reference	(3)	(4)	(5)	(6)	(13) [SEER data]	(41)	(9)	(16)	Present study
**Number**	179	262	370	125	35,680	128	756	91	939
**Period**	1993–1996	1998–2005	1991–2010	2001–2010	1983–2011	2005–2015	2009–2014	1986–2020	2011–2017
**Gender ratio**	4:1	3.1:1	2.9:1	2.2:1	1.17:1	1.16:1	1:1.03	1.37:1	2.07:1
**Stage I/II vs. III/IV**	53% vs. 47%	44% vs. 55%	35% vs. 65%	20% vs. 80%	60.9% vs. 39.1%	41.4% vs. 58.6%	47% vs. 53%	60.1% vs. 39.9%	47% vs. 53%
**Age—median (range)**		30 yrs (15–72)	36 yrs (18–75)	25 yrs (12–68)		28.5 yrs	30 yrs	29 yrs	38 yrs (18-99)
**B’ symptoms**	55%	64%	81%	42%	37.3%	35.1%	69%	NA	50%
**Treatment received**	ABVD ± RT [100%]	ABVD ± RT [85%]	ABVD ± RT [61%]	ABVD ± RT [85%]	NA	ABVD [87.5%],	ABVD [93%]	ABVD ± RT 79.5%	ABVD +/- RT 95%
**Response**	CR *^a^*—100%	CR—92%	CR—81%	NA	NA	CR—57%	CR—73%	CR—82%	CR—80.6%
**5-year survival**	EFS—82	FFTF—78.3%	PFS—66.3%	PFS—NA	NA	PFS—37.3 ± 6.9%	NA	NA	EFS—78.24%
	OS—95	OS—86.6%	OS—79.7%	OS—NA		OS—78.9 ± 6.8%			OS—83.63%

a Only CR patient outcome reported.

NA, Not Available.

The gender ratio documented in previous Indian studies before 2000 was 4:1, while the present study showed a change to 2.07:1 reflecting urbanization and changing attitude of society toward female health in the past two decades (1, 18). A higher proportion of patients with B symptoms (50.43%) and advanced stage (53.24%) were noted at initial presentation, again reflecting delay in presentation and diagnosis attributable in part to a deficiency of expertise and awareness among primary care physicians. Secondly, the propensity to implement empiric treatment for tuberculosis, many times implied on fine needle aspiration, contributed to delays in diagnosis causing stage migration before eventual diagnosis was confirmed by biopsy (20–23). Mixed cellularity contrary to nodular sclerosis was the most common histological subtype (60.60%) in this study with similar reports from India and other limited-resource countries (4, 24–27). The higher frequency of mixed cellularity could be more related to higher incidence of EBV infection (25, 28).

The treatment for early-stage patients in India has been 4 cycles of ABVD with or without consolidation radiotherapy. The trend identified in this study was to complete 6 cycles of ABVD where facilities for PET scan and radiotherapy were limited, accounting for this schedule being implemented in 65% of patients with early-stage disease. PET scans were not done for most patients at baseline. The need to perhaps overtreat with 6 cycles was also likely to augment responses and decrease the chance of relapse, keeping in mind the limited resources to salvage recurrences with chemotherapy and stem cell transplant. In this study, 4 cycles without RT were found inferior to 4 cycles with RT or 6 cycles ABVD. While a large number of favorable risk group patients in early-stage disease were treated with 4 cycles of ABVD and consolidative RT or 6 cycles of ABVD similar to the unfavorable group, the impact of prognostic factors on the differences in survival outcomes of two groups could not be discerned.

For advanced-stage CHL varying definitions of early and advanced stage have been used in literature. In this study, all stage II patients were included in the early stage, making direct comparisons between studies difficult (29–31). The standard treatment included 6 cycles of ABVD with or without consolidative RT. A minority were treated with other intensive or less intensive regimens. Patients treated with any modification of ABVD or other combinations in this study resulted in inferior outcomes (79.97% vs. 47.94%, p < 0.0001). In comparison to previous reports from India, more patients in the present study underwent baseline PET CT assessment and mid-cycle 36.57% or at the end of treatment 46.36% (32, 33), a reflection of PET CT scan becoming more accessible and cheaper and perhaps a greater awareness of the prognostic relevance of an interim PET among physicians. As with previous studies, interim response status by PET-CT scan retained its prognostic significance (90.72% vs. 69.30% p < 0.0001) (34, 35). The EFS and OS for advanced-stage CHL were 73.55% and 79.22%, respectively, at a median follow-up of 50 months. This was contrasting with an earlier retrospective analysis of advanced CHL wherein the OS and EFS were 60% and 58%, respectively, at a median follow-up of 28 months (36). Perhaps the larger numbers and longer follow-up data in the current dataset provided a better outcome.

Looking at the outcomes in both early- and advanced-stage CHL, the overall response rate of 93.67%, 5-year EFS of 78.24%, and OS of 83.63% reported in this study was comparable to previously reported data from Indian as well as Western studies (37–39), but better than some other low-income countries (9, 17). This disparity in results from other low-income nations could be due to differences in methodology, population included, and data gathering approach. However, despite efforts by treating physicians, improving financial conditions and awareness, there was a significant drop in adherence to follow-up especially in the first 2 years after completion of treatment. This has remained constant over the past decade translating into delayed identification of relapses and long-term sequelae adding to morbidity and mortality rates in CHL post therapy. The collaborating institutes understand the need to put in more effort and make tailored strategies to improve adherence to routine follow-ups after completion of treatment.

At relapse, majority of patients (60.0%) received platinum-based salvage chemotherapy. In contrast to Western counterparts (16, 40, 41), only a very small number of relapsed/refractory (8%) patients received novel agents in salvage setting prior to transplantation, the main reasons being the high cost of newer drugs, an out-of-pocket payment system, and also a sense of nihilism to undergo high-dose chemotherapy and a hematopoietic stem cell transplant (HDCT and HSCT), resulting in only a quarter of patients, responding to salvage, receiving consolidative HDCT and HSCT.

This retrospective analysis is the largest Indian dataset specifically looking into the clinical presentation and outcomes in patients with CHL receiving a uniform protocol in the first-line setting. The outcomes are comparable with real-world data from other countries. There is a need for a closer look at outcomes in the relapsed setting which could not be identified with conviction from this dataset owing to non-uniformity of treatment, lack of any specific recommended path of treatment, and more importantly loss to follow-up issues. The advent of more centers offering HDCT and HSCT and increasing availability of immunotherapy with decreasing costs may change the outlook and outcomes in relapsed CHL patients in India.

## Conclusion

OncoCollect Registry was an initiative aimed at analyzing real-world data in order to understand the lacunae in care and improve and modify treatment in future. This study being a retrospective analysis of captured data is less likely to have the inherent bias of a traditional retrospective chart review study. The survival in patients completing treatment is comparable to that of CHL treated in the developed countries. Real-world data are important in understanding outcomes of current standard practices. It is an important reference in initiating changes like de-escalating strategies in early-stage CHL, to reduce long-term toxicity without compromising outcomes and escalating therapy in high-risk patients. In the relapsed/refractory setting, the outcomes of this study are encouraging and recommend that a second chance for cure must be offered to these patients.

## Data Availability Statement

The original contributions presented in the study are included in the article/supplementary material. Further inquiries can be directed to the corresponding author.

## Author Contributions

Concept and design: DB, RNa, HM, SR, RNi. Literature search: DB, HM, RNa, SR. Clinical management: all clinicians mentioned in the contributors list except RNi. Data acquisition, data analysis: RNi, Institute IT Teams, clinical coordinators. Manuscript preparation: DB, RNa, SR, HM, RNi. Manuscript editing: SR, SS, HM, SGR, RNi. Manuscript review: SR, HM, RNa, RNi. IA. Guarantors: DB, RNi. All authors contributed to the article and approved the submitted version.

## Conflict of Interest

Author DB is employed by Rajiv Gandhi Cancer Institute & Research Centre. GSR is a consultant in Kumaran Hospital Private Ltd.

The remaining authors declare that the research was conducted in the absence of any commercial or financial relationships that could be construed as a potential conflict of interest.

## Publisher’s Note

All claims expressed in this article are solely those of the authors and do not necessarily represent those of their affiliated organizations, or those of the publisher, the editors and the reviewers. Any product that may be evaluated in this article, or claim that may be made by its manufacturer, is not guaranteed or endorsed by the publisher.

## References

[1] Shanta V Sastri DV Sagar TG Sasikala K Krishnamurthi S. A Review of Hodgkin’s Disease at the Cancer Institute, Madras. Clin Oncol (1982) 8(1):5–15. 7075045

[2] Chandi L Kumar L Kochupillai V Dawar R Singh R. Hodgkin’s Disease: A Retrospective Analysis of 15 Years Experience at a Large Referral Center. Natl Med J Ind (1998) 11:212–7. 10997167

[3] Laskar S Gupta T Vimal S Muckaden MA Saikia TK Pai SK. Consolidation Radiation After Complete Remission in Hodgkin’s Disease Following Six Cycles of Doxorubicin, Bleomycin, Vinblastine and Dacarbazine Chemotherapy: Is There a Need? L Clin Oncol (2004) 22:62–8. doi: 10.1200/JCO.2004.01.021 14657226

[4] Ganessan P Kumar L Raina V Sharma A Bakshi S Sreenivas V. Hodgkin Lymphoma- Long Term Outcomes: An Experience From a Tertiary Care Center in North India. Ann Hematol (2011) 90:1153–60. doi: 10.1007/s00277-011-1262-8 21625999

[5] Maddi RN Linga VG Iyer KK Chowdhary JS Gundeti S Digumarti R. Clinical Profile and Outcomes of Adult Hodgkin Lymphoma. Experience From a Tertiary Care Institute. In J Med Pediatr Oncol (2015) 36(4):255–60. doi: 10.4103/0971-5851.171550 PMC4711225 26811596

[6] Yadav BS Sharma SC Malhotra P Prakash G. Combined Modality Treatment: Outcome in Patients With Hodgkin’s Lymphoma. J Cancer Res Thera (2020) 16(1):1–6. doi: 10.4103/jcrt.JCRT_465_17 32362601

[7] Seshachalan A Karpurmath SV Rathnam K Ganapathi R Janarthinakani M Prasad K. Does Interim PET Scan After 2 Cycles of ABVD Predict Outcomes in Hodgkin Lymphoma? Real World Evidence. J Glob Oncol (2019) 05:1–13. doi: 10.1200/JGO.19.00179 PMC6939745 31834832

[8] Chatenoud L Bertuccio P Bosetti C Rodriguez T Levi F Negri E. Hodgkin’s Lymphoma Mortality in the Americas, 1997-2008: Achievements and Persistent Inadequacies. Int J Cancer (2013) 133(3):687–94. doi: 10.1002/ijc.28049 23335127

[9] Biasoli I Castro N Delamain M Silveira T Farley J Simões BP. Treatment Outcomes for Hodgkin Lymphoma: First Report From the Brazilian Prospective Registry. Hematol Oncol (2018) 36(1):189–95. doi: 10.1182/asheducation-2009.1.523 28643458

[10] Girinsky T Specht L Ghalibafian M Edeline V Bonniaud G van der Maazen R. The Conundrum of Hodgkin Lymphoma Nodes: To be or Not to be Included in the Involved Node Radiation Fields. The EORTC-GELA Lymphoma Group Guidelines. Radiother Oncol (2008) 88(2):202–10. doi: 10.1016/j.radonc.2008.05.012 18555548

[11] Hoppe RT Advani RH Ai WZ Ambinder RF Aoun P Armand P. NCCN Guidelines Insights: Hodgkin Lymphoma, Version 1.2018. J Natl Compr Canc Netw (2018) 16(3):245–54. doi: 10.6004/jnccn.2018.0013 29523663

[12] Eghbali H Raemaekers J Carde P EORTC Lymphoma Group. The EORTC Strategy in the Treatment of Hodgkin’s Lymphoma. Eur J Haematol Suppl (2005) 66):135–40. doi: 10.1111/j.1600-0609.2005.00467.x 16007882

[13] Hasenclever D Diehl V. A Prognostic Score for Advanced Hodgkin’s Disease. International Prognostic Factors Project on Advanced Hodgkin’s Disease. N Engl J Med (1998) 339:1506–14. doi: 10.1056/NEJM199811193392104 9819449

[14] Cheson BD Beate F Mallik EJ Gascoyne RD Specht L Horning SJ. Revised Response Criteria for Malignant Lymphoma. JCO (2007) 25(5):579–86. doi: 10.1200/JCO.2006.09.2403 17242396

[15] Koshy M Fairchild A Son CH Mahmood U. Improved Survival Time Trends in Hodgkin’s Lymphoma. Cancer Med (2016) 5(6):997–1003. doi: 10.1002/cam4.655 26999817 PMC4924356

[16] Jaime-Pérez JC Gamboa-Alonso CM Padilla-Medina JR Jiménez-Castillo RA Olguín-Ramírez LA Gutiérrez-Aguirre CH. High Frequency of Primary Refractory Disease and Low Progression-Free Survival Rate of Hodgkin’s Lymphoma: A Decade of Experience in a Latin American Center. Rev Bras Hematol e Hemoter (2017) 39:325–30. doi: 10.1016/j.bjhh.2017.08.001 PMC5693277 29150104

[17] Sánchez-Valledor LF Habermann TM Murrieta-Alvarez I Córdova-Ramírez AC Rivera-Álvarez M León-Peña A. Long-Term Results of the Treatment of Hodgkin’s Lymphoma in a Resource-Constrained Setting: Real-World Data From a Single Center. World J Clin Oncol (2021) 12(9):800. doi: 10.5306/wjco.v12.i9.800 34631443 PMC8479346

[18] Talvalkar GV Sampat MB Gangadharan P. Hodgkin’s Disease in Western India: Review of 1082 Cases. Cancer (1982) 50(2):353–9. doi: 10.1002/1097-0142(19820715)50:2<353::AID-CNCR2820500232>3.0.CO;2-# 7083142

[19] World Health Organization. Global Tuberculosis Report 2020. Geneva: World Health Organization (2020). Available at: https://www.who.int/publications/i/item/9789240013131.

[20] Central TB Division. India TB Report 2021. New Delhi: National Health Mission (2021). Available at: https://tbcindia.gov.in/index1.php?lang=1&level=1&sublinkid=4160&lid=280.

[21] Singh S Kumar S. Tuberculosis in India: Road to Elimination. Int J Prev Med (2019) 10:114. doi: 10.4103/ijpvm.IJPVM_492_17 31360361 PMC6592106

[22] Dinshaw KA Advani SH Gopal R Nair CN Talvalkar GV Gangadharan P. Management of Hodgkin’s Disease in Western India. Cancer (1984) 54(7):1276–82. doi: 10.1002/1097-0142(19841001)54:7<1276::AID-CNCR2820540708>3.0.CO;2-E 6467152

[23] Dinand V Dawar R Arya LS Unni R Mohanty B Singh R. Hodgkin’s Lymphoma in Indian Children: Prevalence and Significance of Epstein-Barr Virus Detection in Hodgkin’s and Reed-Sternberg Cells. Eur J Cancer (2007) 43(1):161–8. doi: 10.1016/j.ejca.2006.08.036 17113770

[24] Vashisht S Aikat BK. Hodgkin’s Disease (Retrospective Study of 119 Cases). Indian J Cancer (1973) 10(3):263–79. 4785877

[25] Canellos GP Anderson JR Propert KJ Nissen N Cooper MR Henderson ES. Chemotherapy of Advanced Hodgkin’s Disease With MOPP, ABVD, or MOPP Alternating With ABVD. N Engl J Med (1992) 327(21):1478–84. doi: 10.1056/NEJM199211193272102 1383821

[26] Karnik S Srinivasan B Nair S. Hodgkin’s Lymphoma: Immunohistochemical Features and its Association With EBV LMP-1. Experience From a South Indian Hospital. Pathology (2003) 35(3):207–11. doi: 10.1080/0031302031000123164 14506963

[27] Korula A Devasia AJ Kulkarni U Abubacker FN Lakshmi KM Abraham A. Impact of Imaging Modality on Clinical Outcome in Hodgkin Lymphoma in a Resource Constraint Setting. Br J Haematol (2020) 188(6):930–4. doi: 10.1111/bjh.16289 31811734

[28] Eich HT Diehl V Görgen H Pabst T Markova J Debus J. Intensified Chemotherapy and Dose-Reduced Involved-Field Radiotherapy in Patients With Early Unfavorable Hodgkin’s Lymphoma: Final Analysis of the German Hodgkin Study Group HD11 Trial. J Clin Oncol (2010) 28(27):4199–206. doi: 10.1200/JCO.2010.29.8018 20713848

[29] Fermé C Eghbali H Meerwaldt JH Rieux C Bosq J Berger F. EORTC-GELA H8 Trial. Chemotherapy Plus Involved-Field Radiation in Early-Stage Hodgkin’s Disease. N Engl J Med (2007) 357(19):1916–27. doi: 10.1056/NEJMoa064601 17989384

[30] Allemani C Sant M De Angelis R Marcos-Gragera R Coebergh JW EUROCARE Working Group. Hodgkin Disease Survival in Europe and the U.S.: Prognostic Significance of Morphologic Groups. Cancer (2006) 107(2):352–60. doi: 10.1002/cncr.21995 16770772

[31] Stefan DC Stones D. How Much Does it Cost to Treat Children With Hodgkin Lymphoma in Africa? Leuk Lymphoma (2009) 50(2):196–9. doi: 10.1080/10428190802663205 19197725

[32] Ganesan P Rajendranath R Kannan K Radhakrishnan V Ganesan TS Udupa K. Phase II Study of Interim PET-CT-Guided Response-Adapted Therapy in Advanced Hodgkin’s Lymphoma. Ann Oncol (2015) 26(6):1170–4. doi: 10.1093/annonc/mdv077 25701453

[33] Straus DJ Jung SH Pitcher B Kostakoglu L Grecula JC Hsi ED. CALGB 50604: Risk-Adapted Treatment of Nonbulky Early-Stage Hodgkin Lymphoma Based on Interim PET. Blood (2018) 132(10):1013–21. doi: 10.1182/blood-2018-01-827246 PMC6128083 30049811

[34] Engert A Plütschow A Eich HT Lohri A Dörken B Borchmann P. Reduced Treatment Intensity in Patients With Early-Stage Hodgkin’s Lymphoma. N Engl J Med (2010) 363(7):640–52. doi: 10.1056/NEJMoa1000067 20818855

[35] Jain H Sengar M Nair R Menon H Laskar S Shet T. Treatment Resultsin Advanced Stage Hodgkins Lymphoma: A Retrospective Study. J Postgrad Med (2015) 61(2):88–91. doi: 10.4103/0022-3859.150446 25766339 PMC4943438

[36] Brenner H Gondos A Pulte D Shet T. Survival Expectations of Patients Diagnosed With Hodgkin’s Lymphoma in 2006-2010. Oncologist (2009) 14(8):806–13. doi: 10.1634/theoncologist.2008-0285 19648314

[37] Brenner H Gondos A Pulte D. Ongoing Improvement in Long-Term Survival of Patients With Hodgkin Disease at All Ages and Recent Catch-Up of Older Patients. Blood (2008) 111(6):2977–83. doi: 10.1182/blood-2007-10-115493 18096762

[38] Verdecchia A Francisci S Brenner H Gatta G Micheli A Mangone L. Recent Cancer Survival in Europe: A 2000-02 Period Analysis of EUROCARE-4 Data. Lancet Oncol (2007) 8(9):784–96. doi: 10.1016/S1470-2045(07)70246-2 17714993

[39] Uncu Ulu B Dal MS Yönal Hindilerden İ Akay OM Mehtap Ö Büyükkurt N. Brentuximab Vedotin and Bendamustine: An Effective Salvage Therapy for Relapsed or Refractory Hodgkin Lymphoma Patients. J Chemother (2021) 4:1–9. doi: 10.1080/1120009X.2021.1976912 34514960

[40] Taçyıldız N Tanyıldız HG Ünal E Dinçaslan H Asarcıklı F Aksoy BA. A Targeted Salvage Therapy With Brentuximab Vedotin in Heavily Treated Refractory or Relapsed Pediatric Hodgkin Lymphoma Patients Before and After Stem Cell Transplantation. Turk J Pediatr (2019) 61(5):671–6. doi: 10.24953/turkjped.2019.05.005 32104998

[41] Zinzani PL Chen R Armand P Johnson NA Brice P Radford J. Pembrolizumab Monotherapy in Patients With Primary Refractory Classical Hodgkin Lymphoma Who Relapsed After Salvage Autologous Stem Cell Transplantation and/or Brentuximab Vedotin Therapy: KEYNOTE-087 Subgroup Analysis. Leuk Lymphoma (2020) 61(4):950–4. doi: 10.1080/10428194.2019.1702178 PMC7319053 31905294

